# Sequential Intra-articular Migration of Broken Circumferential Wire Fragments Following Patellar Surgery: A Case Report

**DOI:** 10.7759/cureus.111547

**Published:** 2026-06-26

**Authors:** Yudai Yano, Kimitaka Nakamura, Takahiro Hamada, Akihiko Inokuchi, Teiyu Izumi, Ryuta Imamura, Yuki Hyodo, Maki Noguchi, Sho Hirata, Takeshi Arizono

**Affiliations:** 1 Orthopaedic Surgery, Kyushu Central Hospital, Fukuoka, JPN

**Keywords:** cerclage compression wiring, chondral injury, intra-articular migration, patellar fracture, wire breakage

## Abstract

Cerclage compression wiring (CCW) is a standard surgical procedure for transverse patellar fractures. While hardware breakage is a common complication, the intra-articular migration of broken wires is rare. We report the case of a 79-year-old female with a four-part transverse patellar fracture (AO classification: 34C3) who underwent open reduction and internal fixation using CCW with additional circumferential wiring. Although the initial postoperative course was favorable, the circumferential wire fractured one year postoperatively, and patellar non-union was also confirmed at that time. Between 15 and 17 months postoperatively, further breakages and sequential migrations occurred one after another; these fragments migrated into the medial joint space and the posteromedial aspect of the fibular head, eventually leading to mechanical symptoms and limited range of motion. Surgical retrieval using a combined arthroscopic and open approach identified a linear cartilage lesion on the medial femoral condyle, likely representing mechanical abrasion caused by the migrated fragment. The deep placement of the circumferential wire potentially contributed to mechanical wear and subsequent fracture. Broken metal wires carry a risk of migration, and their presence within the joint space poses a risk of chondral injury. Improving surgical techniques, such as avoiding excessively deep hardware placement, and considering the optimal choice of fixation materials remain important challenges in preventing such hardware-related complications.

## Introduction

Cerclage compression wiring (CCW) with additional circumferential wiring is a standard surgical procedure for transverse patellar fractures. While hardware-related complications, such as wire breakage, are frequently encountered in clinical practice, the intra-articular migration of a broken circumferential wire is exceedingly rare. To our knowledge, only a limited number of cases have been reported in the literature. We herein report a case in which a broken circumferential wire migrated into the knee joint, leading to mechanical symptoms and associated chondral injury.

## Case presentation

**Figure 1:**
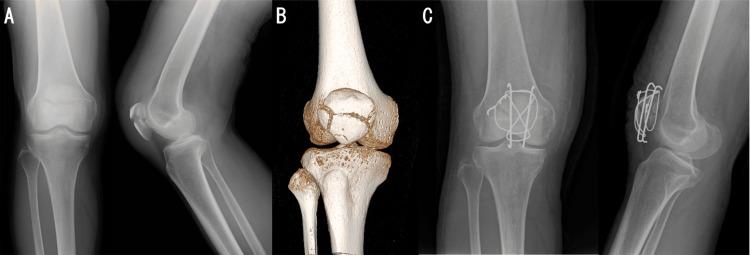
Imaging findings of the initial injury and primary surgery A: Preoperative plain radiographs (anteroposterior (AP) and lateral views) showing a four-part patellar fracture. B: Preoperative 3D computed tomography reconstruction highlighting the multi-fragmentary fracture pattern. C: Postoperative radiographs (AP and lateral views) after the primary ORIF using the CCW technique with additional circumferential wiring. CCW, cerclage compression wiring; ORIF, open reduction and internal fixation.

A 79-year-old female presented to our hospital with a right knee injury after a fall at home. Her medical history was unremarkable except for short-term memory impairment. Plain radiographs and computed tomography scans revealed a four-part transverse patellar fracture (AO classification: 34C3), involving a third fragment on the lateral side and a small fragment on the medial side. Additionally, a subtle cortical irregularity was noted on the medial femoral condyle (Figures 1A, 1B).

Three days after the injury, the patient underwent open reduction and internal fixation using the CCW technique with additional circumferential wiring. The proximal and distal fragments were reduced and secured using two Kirschner wires (K-wires), and a 1.0-mm diameter stainless steel wire was tensioned in a figure-of-eight fashion over the anterior aspect of the patella. Subsequently, an additional circumferential wire (1.0-mm diameter stainless steel wire) was placed to compress the lateral and medial fragments (Figure 1C).

Postoperatively, the patient was permitted to begin ambulation with a knee brace on day 1, and range-of-motion (ROM) exercises were initiated on day 7. The postoperative course was uneventful, and she was transferred to a rehabilitation facility on day 17 to continue physical therapy.

The patient discontinued follow-up visits because the initial postoperative course was favorable. However, one year after surgery, she returned to our hospital complaining of right knee pain. Physical examination revealed joint effusion and tenderness at the medial aspect of the knee; notably, no tenderness was elicited over the patellar non-union site. The ROM was preserved at 0°-130° without motion-induced pain. Arthrocentesis yielded clear, straw-colored fluid.

**Figure 2:**
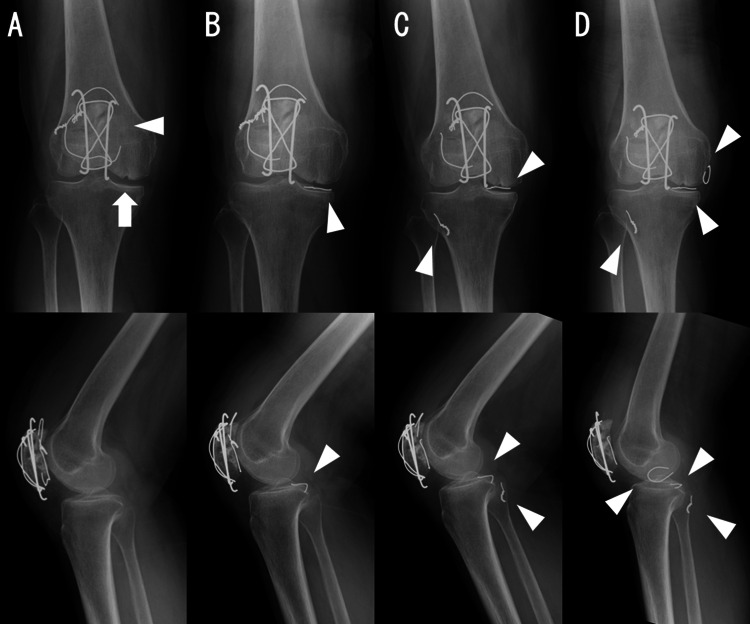
Sequential radiographs showing wire breakage and migration A: Radiographs at 12 months postoperatively (AP and lateral views). The patellar non-union and breakage of the circumferential wire are visible (arrowhead). Note the subchondral bone depression on the medial femoral condyle (arrow). B-D: Serial radiographs showing the progressive migration of wire fragments at 15 months (B), 16 months (C), and 17 months (D) postoperatively (arrowhead). AP, anteroposterior.

Radiographs revealed a non-union of the patellar fracture and a breakage of the circumferential wire. Although the broken fragments showed displacement at the breakage sites, they remained in situ without evidence of migration. Additionally, a depression was observed on the medial femoral condyle, at the same site where the initial cortical irregularity had been noted (Figure 2A). Based on these findings, we attributed the knee pain to medial osteoarthritis, possibly secondary to osteonecrosis or a subchondral insufficiency fracture of the knee (SIFK), rather than the patellar non-union. Although hardware removal was initially proposed, the patient remained asymptomatic regarding the patellar non-union and did not wish to undergo surgery. Therefore, we continued conservative treatment for the right knee osteoarthritis and initiated low-intensity pulsed ultrasound therapy for the patellar non-union [1].

At 15 months postoperatively, radiographs identified the migration of a broken wire fragment into the posterior aspect of the medial joint space (Figure 2B). Despite this finding, the patient remained asymptomatic, with a preserved ROM of 0°-130°. Although surgical removal was proposed, the patient declined due to the absence of symptoms, and observation was continued. At 16 months postoperatively, further breakage of the wire was observed, with another fragment migrating to the posteromedial aspect of the fibular head (Figure 2C). By 17 months postoperatively, ROM became slightly limited to 0°-120°, with the onset of motion-induced pain. Radiographs revealed the migration of an additional broken wire fragment into the medial aspect of the knee joint (Figure 2D). Given the development of these mechanical symptoms, the patient opted for surgical removal.

**Figure 3:**
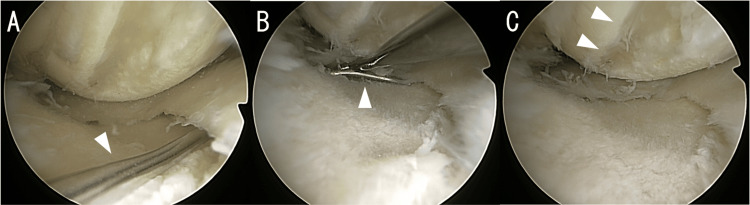
Intraoperative arthroscopic findings A: Identification of the wire fragment in the medial joint space (arrowhead) B: Mobilization of the incarcerated fragment posterior to the medial tibia using a probe inserted beneath the posterior horn of the medial meniscus (arrowhead) C: A linear cartilage lesion on the medial femoral condyle (arrowhead), suggestive of mechanical "windshield-wiper" injury

**Figure 4:**
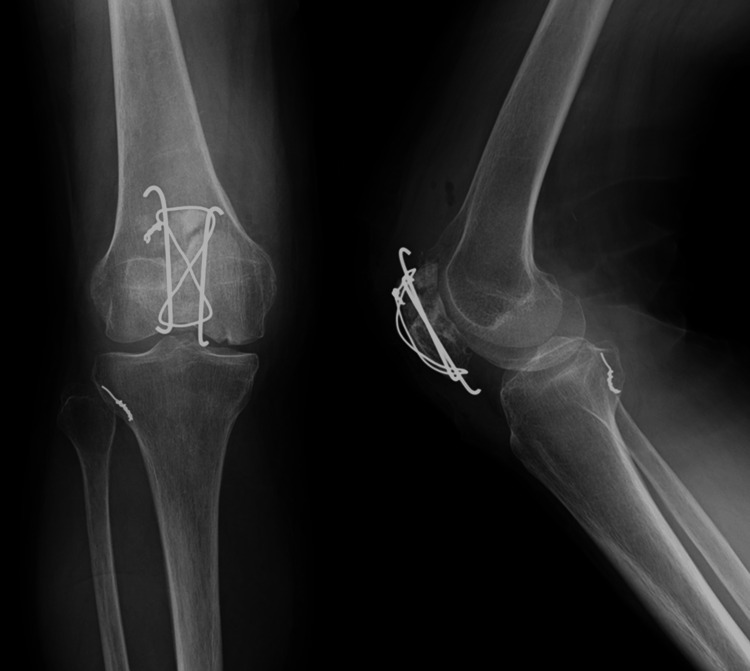
Radiographs (AP and lateral views) after wire retrieval AP, anteroposterior.

Surgical retrieval was performed using a combined arthroscopic and open approach. First, arthroscopy was conducted, and the wire fragment identified in the medial joint space was retrieved (Figure 3A). Another fragment located posterior to the medial tibia was not initially visible by arthroscopy alone. However, it was successfully mobilized by inserting a probe beneath the posterior horn of the medial meniscus and subsequently removed (Figure 3B). Following the retrieval, a detailed inspection of the joint surface revealed a linear cartilage lesion on the medial femoral condyle (Figure 3C). A subchondral bone depression was also observed in the same area, consistent with the preoperative diagnosis of SIFK. Given its distinct linear morphology, this cartilage lesion was considered to potentially represent mechanical abrasion caused by the migrated wire fragment, superimposed on the pre-existing degenerative changes. Furthermore, arthroscopic examination confirmed residual instability at the patellar non-union site during knee motion. Following the arthroscopy, an open approach was used for further wire removal. Due to the confirmed instability of the non-union site, the original CCW (K-wires and figure-of-eight wire) was left in situ to provide structural support, while the remaining broken circumferential wires on the lateral side were removed through the previous surgical incision. The wire fragment located posteromedial to the fibular head was also left in situ, considering its stable position throughout the preoperative follow-up and the potential risk of neurovascular injury associated with aggressive retrieval. To further stabilize the non-union, circumferential cerclage was performed using two strands of no. 2 high-strength non-absorbable suture. Finally, improved stability of the non-union site was confirmed arthroscopically before concluding the procedure (Figure 4).

Postoperatively, full weight-bearing was permitted without restrictions, and the motion-induced pain improved. At the latest outpatient follow-up, radiographs confirmed that the retained wire fragment remained stable without migration, and no neurological deficits were observed.

## Discussion

CCW using K-wires and metal wires remains a standard surgical procedure for patellar fractures. Although hardware failure is a recognized complication, intra-articular migration of a broken wire fragment is extremely rare. To the best of our knowledge, only a limited number of such cases have been reported in the literature (Table 1) [2,3]. Wang et al. hypothesized that such intra-articular migration is infrequent because a wire fragment would need to penetrate multiple soft tissue layers, including the joint capsule, to enter the joint space [3]. In the present case, however, multiple broken fragments were found to have bypassed these anatomical barriers and entered the joint space, leading to mechanical symptoms.

**Table 1:** The previously reported cases of intra-articular migration of broken wire fragments

Case report	Patient age (years)/sex	Primary surgery	Migration route	Joint affected	Outcome
Sharma et al., 2016 [2]	51/male	Tension band wiring with cerclage wiring	Unknown	Knee	Successful surgical removal via arthroscopy; symptoms resolved
						Wang and Lee, 2013 [3]	61/male	Tension band wiring	Through the pseudarthrosis line of the patellar fracture	Knee	Successful removal of the wire fragment; knee locking resolved

Risk factors for hardware failure generally include high patient activity levels, improper wire tension, and deep placement of the hardware within the quadriceps tendon [4]. Selçuk et al. reported that factors such as a single tightening site, excessive K-wire length, and a greater distance between the K-wire and the patellofemoral joint surface are associated with hardware failure [5]. In the present case, we believe that a technical error during the initial procedure was a primary contributing factor. The circumferential wire might have been placed too deeply (Figure 1C), potentially traversing the intra-articular space. This deep positioning, coupled with persistent mechanical stress from the patellar non-union, accelerated metal fatigue. Furthermore, the circumferential wiring technique necessitates passing the wire through soft tissues at multiple points. We hypothesize that each of these passage points may have acted as a site for stress concentration or subtle structural damage during insertion, potentially predisposing the wire to fatigue failure at multiple locations. The initial failure of one site likely redistributed the mechanical load to the remaining weakened points, leading to further breakages and subsequent migration of fragments observed over several months. Consequently, these fragments easily migrated into the joint space, subsequently causing mechanical irritation and the observed linear cartilage lesion through the so-called "windshield-wiper effect."

Other reported sites of hardware migration after patellar fixation include the tibia, popliteal fossa, ankle joint, and even the heart [4,6-9]. Previous literature suggests that when wire breakage occurs, removal should be considered to prevent neurovascular complications or distant migration into potentially life-threatening locations. Furthermore, as demonstrated in the present case, broken fragments can migrate sequentially and cause mechanical cartilage attrition even after a period of stability. Therefore, early removal of broken hardware, particularly when fragments are located near or within the joint space, may be worth considering to prevent delayed intra-articular complications. However, the decision for retrieval should be individualized by weighing the risk of further migration against the potential surgical morbidity associated with the procedure. In the present case, while the intra-articular fragments were retrieved to resolve the mechanical symptoms and prevent further chondral damage, the fragment located posteromedial to the fibular head was left in situ. This decision was based on the fragment’s stable position on serial radiographs and the overall clinical context. Given the patient’s advanced age, cognitive impairment, and relatively low physical activity levels, our primary surgical goal was to alleviate the acute symptoms with minimal invasiveness. Therefore, we prioritized avoiding extensive surgical dissection near the common peroneal nerve for a stable, asymptomatic fragment. Nevertheless, since the risk of further migration cannot be entirely ruled out, diligent long-term follow-up is essential to monitor for any delayed complications.

In recent years, the use of high-strength non-absorbable sutures such as FiberWire (Arthrex, Naples, FL, USA) instead of metal hardware has gained popularity as a means to reduce complications such as hardware failure and skin irritation [10,11]. Noothan et al. reported that high-strength sutures provided functional and radiological outcomes comparable to those of stainless steel wires, with a lower incidence of hardware-related issues [12]. However, the choice of material involves a critical trade-off. Metal wires offer superior radiographic visibility, allowing for the early detection of breakage and subsequent adjustment of postoperative management. In contrast, suture breakage is difficult to identify on conventional imaging, posing a risk of unrecognized loss of fixation. Therefore, the selection between metal and suture should be carefully individualized based on the clinical priorities.

In summary, the clinical course of this case highlights several critical insights for preventing and managing hardware complications associated with circumferential wiring. First, meticulous surgical technique is paramount: avoiding excessively deep hardware placement is essential to prevent wires from traversing the intra-articular space, while care must be taken during wire insertion to minimize structural damage at soft-tissue passage points. Second, the use of high-strength non-absorbable sutures may be considered as an alternative option to minimize the inherent risk of fatigue failure, particularly in circumferential wiring techniques that require passing the wire through soft tissues at multiple points. Finally, a vital lesson from this experience concerns the management of asymptomatic hardware failure. When wire breakage occurs, both clinicians and patients may naturally incline toward conservative observation due to the initial absence of symptoms. However, as demonstrated in this case, when breakage occurs at multiple locations, the fragments can dislodge, potentially causing irreversible secondary damage, such as chondral attrition, through delayed sequential migration. Therefore, rather than passive monitoring, prompt and proactive removal of broken hardware is highly recommended to avert complications such as delayed and severe intra-articular damage.

## Conclusions

We experienced a case of patellar fracture in which a broken circumferential wire fragment migrated sequentially into the joint space and was associated with chondral injury. Improving surgical techniques, such as avoiding excessively deep hardware placement, and considering the optimal choice of fixation materials remain important challenges in preventing such hardware-related complications.
